# Effects of Hydraulic Loading Rate on Nutrients Removal from Anaerobically Digested Swine Wastewater by Multi Soil Layering Treatment Bioreactor

**DOI:** 10.3390/ijerph15122688

**Published:** 2018-11-29

**Authors:** Junyuan Guo, Yuling Zhou, Yijin Yang, Cheng Chen, Jiajing Xu

**Affiliations:** College of Resources and Environment, Chengdu University of Information Technology, Chengdu 610225, China; yuzijiang626@163.com (Y.Z.); jinyiyang@163.com (Y.Y.); 3160202006@cuit.edu.cn (C.C.); xujiajingkk@163.com (J.X.)

**Keywords:** multi soil layering treatment (MSL), anaerobically digested swine wastewater (ADSW), ammonia-N removal, total nitrogen (TN) removal, total phosphorus (TP) removal

## Abstract

A multi soil layering (MSL) treatment bioreactor was developed aiming at nutrients removal from anaerobically digested swine wastewater (ADSW). The start-up of the MSL bioreactor and its performance in nutrients removal at different hydraulic loading rate (HLR) were investigated. Results showed that the MSL bioreactor was successfully started up after operation for 28 days, and at this time, the removal efficiencies of ammonia-N, total nitrogen (TN) and total phosphorus (TP) in the ADSW reached 63.6%, 58.5%, and 46.5%, respectively. The MSL bioreactor showed a stable performance during the whole working process with varying HLR from 80 to 200 L/(m^2^·day). Maximum removal efficiencies of ammonia-N, TN and TP were obtained at 160 L/(m^2^·day), and was appeared as 94.2%, 94.4%, and 92.5%, respectively. It was worth noting that iron scraps were the key factor that enhanced the independent capability of the MSL bioreactor in TP removal, because there was only 21.4–25.8% of the TP was removed when the MSL bioreactor run with no iron addition.

## 1. Introduction

Nowadays, swine wastewater was considered as one of the biggest culprits for the severe agricultural non-point pollution, because of its high concentration of ammonia, organic pollutants, and phosphorus were not managed properly [1,2]. Although anaerobic digestion was thoroughly investigated and applied technology in the treatment of the swine wastewater all over the world, residual nutrients were still considerable in the liquid named “ADSW” (anaerobically digested swine wastewater) [3]. In recent years, land treatment systems were becoming more and more popular in the treatment of the ADSW [4].

The MSL (multi soil layering) bioreactor was known as an effective soil-based technology in the conventional poorly functioning sewage treatment via the enhancing inherent ability of soil [5]. This system has been tested to remove pollutants from polluted river water [6] and domestic wastewater [7], but there was no research related to nutrients removal from swine wastewater. Why can nutrients be effectively removed by the MSL bioreactor from ADSW? As known, the MSL bioreactor was a biofilm reactor that filled with two kinds of media—one was the soil mixture block (SMB) composed of soil and some organic materials, such as sawdust and charcoal, with a ratio of 85:15 by dry weight, and another was the zeolite and iron layers. In case of natural ventilation or manual intervention ventilation, aerobic environments were formed in the zeolite layers due to its porous structures, when the ADSW flowed through the MSL bioreactor, ammonia-N was adsorbed by the zeolite and the nitrifying bacteria were then grew fast. At the same time, anoxic environments were formed in the SMB after who was soaked within the ADSW, and the denitrifying bacteria were gradually grown [7]. Therefrom, ammonia-N was transformed to NO_3_^−^ first in the aerobic environments and then to gaseous nitrogen in the anoxic environments [8]. In the transformation process of NO_3_^−^ to gaseous nitrogen in the anoxic environments in SMB, the organic materials (e.g., sawdust and charcoal) can be used as extra carbon sources for denitrifying bacteria [9]. The role of the iron that was added in the MSL bioreactor was enhancing TP (total phosphorus) removal by the chemical reaction between Fe^3+^ and PO_4_^3−^ [10]. It can clearly be seen that the MSL bioreactor will be an option in the removal of nutrients from the ADSW.

Aims of this study were (1) to construct a MSL bioreactor to remove nutrients simultaneously from the ADSW, (2) to investigate the effects of HLR (hydraulic loading rate) on the removal of nutrients, and (3) to discuss the mechanisms that the MSL bioreactor can remove nutrients during its working period.

## 2. Materials and Methods

### 2.1. Swine Wastewater

Swine wastewater for detecting the performance of MSL bioreactor in nutrients removal was obtained from a pig farm at Chengdu city, Sichuan Province, China. In this pig farm, the joint of Up-flow Anaerobic Sludge Bed (USAB) and Sequencing Batch Reactor (SBR) was applied to treat swine wastewater. The swine wastewater mainly including pig manure, pig urine, and pig house flushing water. Concentrations of COD (chemical oxygen demand), ammonia-N,TN (total nitrogen) and TP of the swine wastewater were 5683.4, 645.8, 752.2, and 26.5 mg/L, respectively, which appeared as 1130.5, 682.6, 761.8, and 22.8 mg/L, respectively after treated by the UASB. The pH values of the swine wastewater and the correspondingly ADSW were almost the same, 6.7 and 6.8, respectively.

### 2.2. Experimental Apparatus

**Figure 1:**
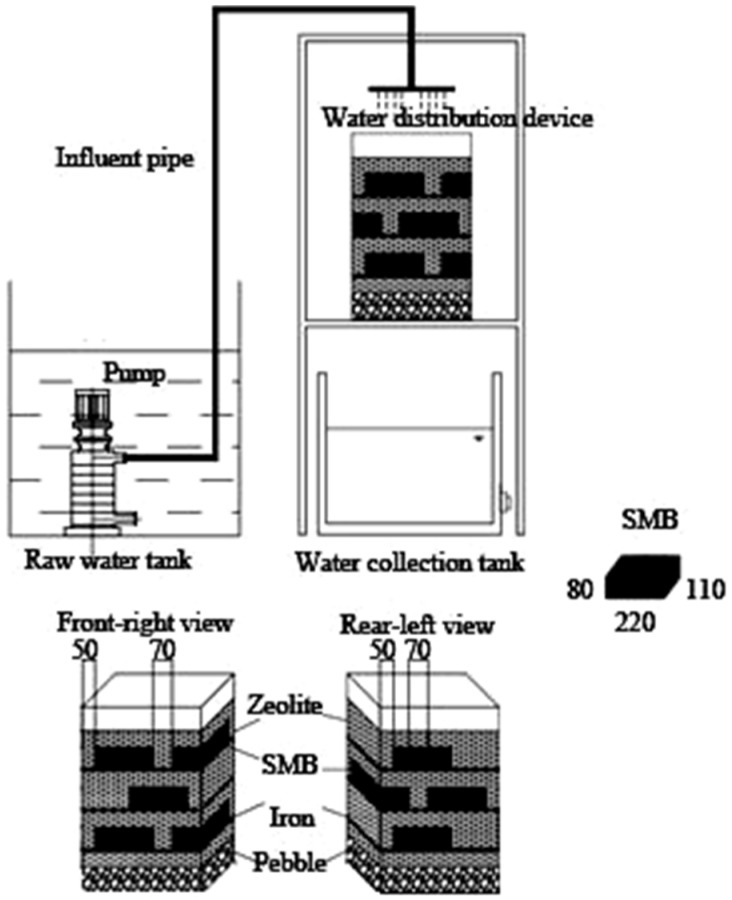
Experimental device and detailed structure of the MSL (multi soil layering treatment) bioreactor.

According to our previous study, the experimental MSL bioreactor was constructed by using a lidless acrylic box filled with different media [11]. Dimensions the MSL bioreactor and the layout of the filter media were showed in Figure 1. The acrylic box was measured as 450 mm × 250 mm × 700 mm, whose bottom was drilled with an aperture area rate of 28%. The aperture was 0.8 mm. From the bottom of the acrylic box, a pebble layer was firstly filled, and then the alternately filling of zeolite layers, iron layers, and SMB layers. Numbers of pebble layers, zeolite layers, iron layers, and soil layers were 1, 4, 3, and 3, respectively. Natural zeolite was selected to build the zeolite layers, and the SMB with a size of 220 mm × 110 mm × 80 mm was the mixture of clayey soil and sawdust (with a ratio of 85:15 by dry weight). The iron scraps were lathe iron cutting scraps obtained from the metal technology practice base of Chengdu University of Information Technology. After being treated by the MSL bioreactor, the water was allowed to naturally flow into the collection tank through the aperture bottom.

### 2.3. ADSW Treatment Process

To start up the MSL bioreactor, firstly, the mixture of the ADSW and the sludge from the UASB of the pig farm, with a volume ratio of 2:1, was pumped and dispersed into the filter media to seed the MSL bioreactor. After one week, the mixture of the ADSW and the sludge was replaced by the same volume of ADSW alone to continuously seed the MSL bioreactor. During the start-up process, the influent HLR was kept at approximately 50 L/(m^2^·day). After start up the MSL bioreactor successfully, effects of the HLR on the MSL bioreactor in nutrients removal were investigated. During the experiment, the mean HLR was set at 80, 120, 160, and 200 L/(m^2^·day) in steps, and at each HLR condition, after 7 day’s stable run of the MSL bioreactor at 50 L/(m^2^·day), the HLR was began changed and water samples were continuously taken and analyzed for 14 days. In addition, the effects of inlet ammonia-N concentration on ammonia-N removal by the MSL were investigated as well.

### 2.4. Analytic Methods

Analysis methods of COD, ammonia-N, NO_3_^−^, TN, and TP were summarized as follows: COD using the potassium dichromate method, ammonia-N using the Nessler’s reagent colorimetric method, NO_3_^−^ by the ultraviolet spectrophotometry method, TN by the potassium persulfate oxidation-ultraviolet spectrophotometry method, and TP using the potassium persulfate digestion colorimetric method [12]. Water pH was measured using pH meter (PHS-3C). The microstructure of the zeolites before and after start-up of the MSL bioreactor was characterized with environmental scanning electron microscopy (Quanta 200, FEI Ltd., Eindhoven, The Netherlands).

## 3. Results and Discussion

### 3.1. Start-Up of the MSL

**Figure 2:**
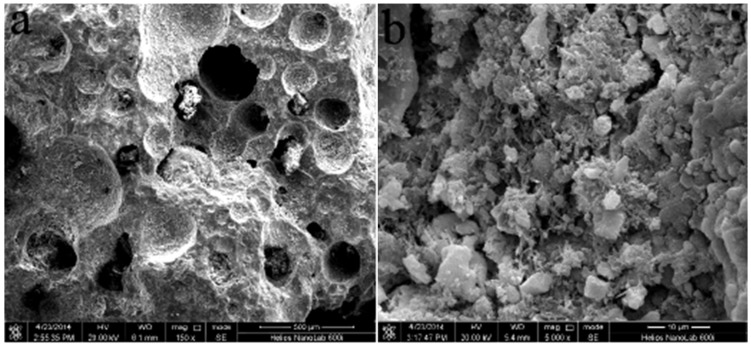
SEM images of the zeolites in the MSL (multi soil layering treatment) bioreactor before start-up (**a**) and after 14 days of start-up (**b**).

As shown in Figure 2a, before start-up of the MSL bioreactor, the raw zeolite in the MSL showed an irregular void structure, which was beneficial to the growth of microorganisms and formed the biofilm. After seven days of start-up, a change of the zeolite color from grayish white to brown was discovered by the naked eye, and suggested that there were microorganisms growing on the surface of the zeolite. As shown in Figure 2b, after 14 days of start-up, a velvet biofilm that had been apparently growing on the surface of the zeolite indicated an efficient biofilm formation in the MSL bioreactor, which can adsorb and degrade ammonia-N and organic pollutants in ADSW [7].

**Figure 3:**
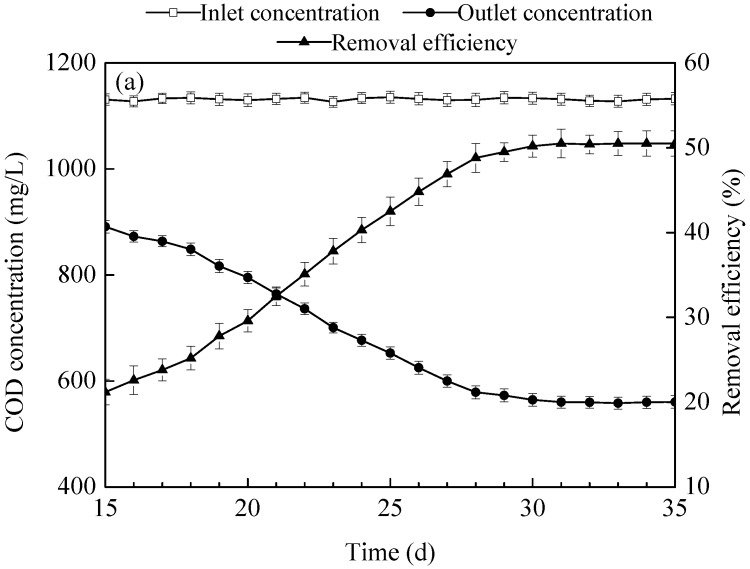

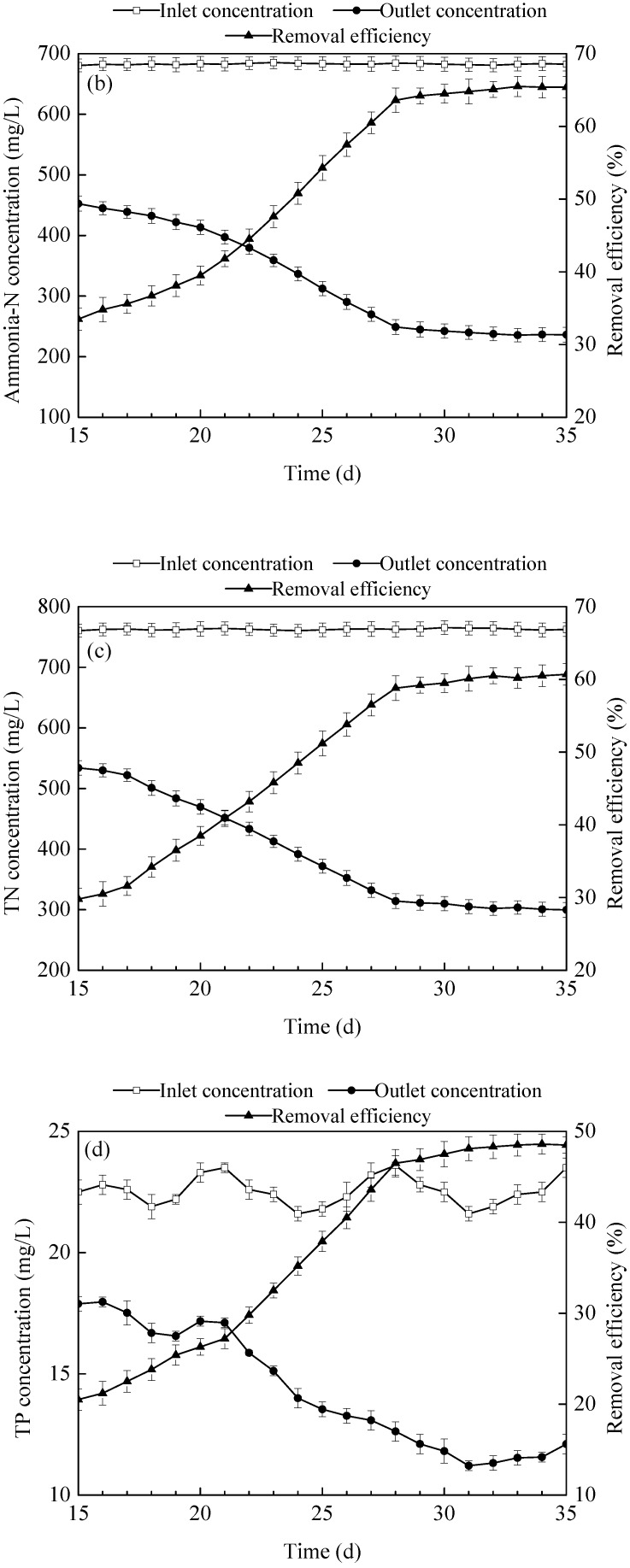
Concentrations and removal efficiencies of COD (chemical oxygen demand) (**a**), ammonia-N (**b**), TN (total nitrogen) (**c**) and TP (total phosphorus) (**d**) during the start-up period of the MSL bioreactor.

From the 15th day onwards, the water qualities in and out the MSL were continuously collected and monitored, which were shown in Figure 3. The removal efficiencies of COD, ammonia-N, TN, and TP was increased from 21.2%, 33.5%, 29.8%, and 20.5% at 15 days to 48.8%, 63.6%, 58.5%, and 46.5% at 28 days, respectively, along with the prolonged time. This is due to the continuous ingestion of organic matter, nitrogen and phosphorus in the ADSW by microorganisms in the MSL bioreactor [11].

At 28–35 days, the removal efficiencies of COD, ammonia-N, TN, and TP can reach 50.5%, 65.4%, 60.7%, and 48.5%, respectively (the corresponding effluent concentrations were lower than 559.6, 236.2, 299.4, and 11.7 mg/L, respectively). The relative deviations of the two monitoring results of COD, ammonia-N, TN, and TP in the effluent were less than 5%, indicated that the MSL bioreactor reached stable operation. This state further illustrated that the biofilm was successfully formed in the MSL bioreactor [11].

### 3.2. Effects of Hydraulic Loading Rate (HLR) on Nutrients Removal

#### 3.2.1. Phosphorus Removal

**Figure 4:**
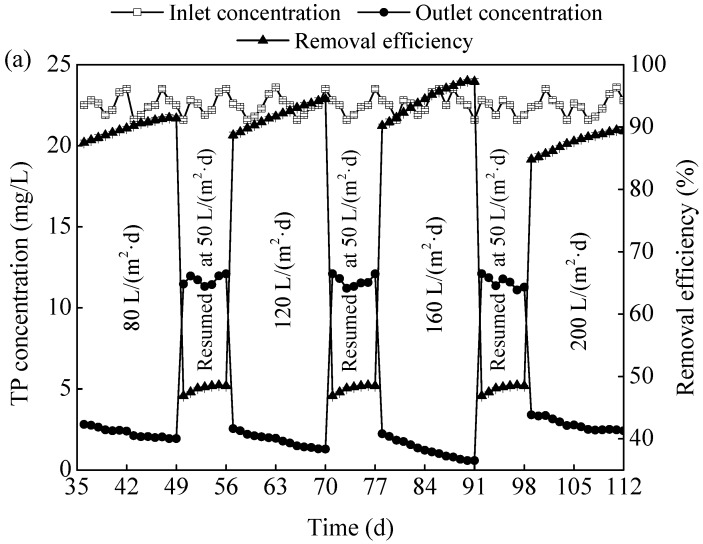

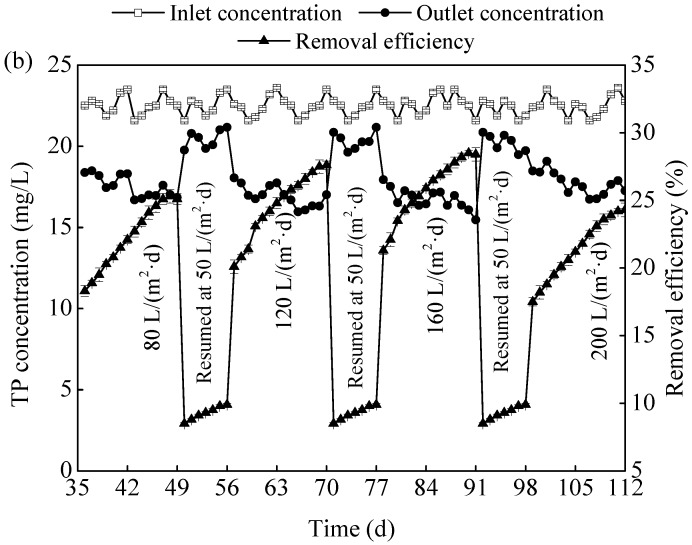
Effects of HLR (hydraulic loading rate) on TP (total phosphorus) removal by the MSL (multi soil layering) with (**a**) and without (**b**) Fe addition.

As shown in Figure 4a, under different HLR from 80 to 200 L/(m^2^·day), 87.6–94.4% of TP on average can be removed from the ADSW, with the final effluent TP concentration kept in the range of 1.3–2.8 mg/L, meaning that the TP in the ADSW can be effectively removed by the MSL bioreactor. The literature has reported that the TP removal in the MSL bioreactor was mainly due to the chemical reaction between Fe^3+^ and PO_4_^3−^, and the adsorption by Fe(OH)_3_ [7,11].

(1) $$\mathrm{Fe}-{2e}^{-}\to {\mathrm{Fe}}^{2+}$$

(2) $${\mathrm{Fe}}^{2+}-e^{-}\to {\mathrm{Fe}}^{3+}$$

(3) $$O_{2}+{2H}_{2}O+{4e}^{-}\to 4{\mathrm{OH}}^{-}$$

(4) $${\mathrm{Fe}}^{3+}+{3\mathrm{OH}}^{-}\to \mathrm{Fe}{\left(\mathrm{OH}\right)}_{3}\;K^{\theta }{}_{\mathrm{sp}}=4.0\times 10^{-38}$$

(5) $${\mathrm{Fe}}^{3+}+{\mathrm{PO}}_{4}{}^{3-}\to {\mathrm{FePO}}_{4}\;K^{\theta }{}_{\mathrm{sp}}=1.3\times 10^{-22}$$

As shown in Equations (1) and (2), in aerobic environment, iron was transformed into Fe^2+^, and then to Fe^3+^, which aids in TP removal through forming FePO_4_ precipitate, at the same time, the reaction O_2_ + 2H_2_O + 4e^−^ → 4OH^−^ (Equation (3)) occurred, a small amount of Fe(OH)_3_ was formed, which can enhance TP removal through coagulation. TP was mainly removed through these two ways.

According to the equilibriums (Equations (4) and (5)), K_sp_^θ^ of the FePO_4_ precipitation equilibrium was higher than Fe^3+^ hydrolysis equilibrium, thus, Fe^3+^ reacted with PO_4_^3−^ prior to OH^−^, thus far, chemical reaction between Fe^3+^ and PO_4_^3−^ was the key way for TP removal [13].

In order to certify the important role of iron in TP removal, a control MSL bioreactor that was run without an iron layer in the same procedure. As shown in Figure 4b, a relatively poor TP removal efficiency of about 21.4–25.8% was obtained at different HLR, which further illustrated that the TP was mainly removed due to the chemical reaction between Fe^3+^ and PO_4_^3−^, and the precipitate would subsequently be adsorbed and/or intercepted by the filter media in the hybrid system. A similar conclusion was reported by Sato et al. (2005) [10].

#### 3.2.2. Ammonia-N Removal

**Figure 5:**
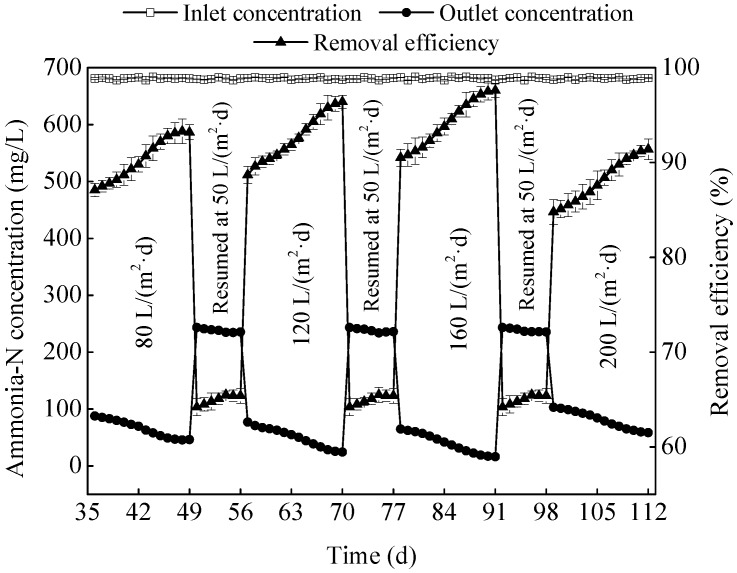
Effects of HLR (hydraulic loading rate) on ammonia-N removal by the MSL (multi soil layering).

Figure 5 clearly showed that the MSL bioreactor can remove 92.6% of ammonia-N from the ADSW at HLR of 120 L/(m^2^·day), and 94.2% at 160 L/(m^2^·day), whereas it was decreased to 88.1% when the HLR was adjusted to 200 L/(m^2^·day). The main reason for the reduction of ammonia-N removal efficiency was that the nitrifying bacteria were at disadvantage in the fierce competition (by the HLR up to 200 L/(m^2^·day)) of living space with heterotrophic bacteria [14]. Apart from this, HLR as high as 200 L/(m^2^·day) shortened the hydraulic retention time (HRT) of the wastewater, which decreased the ammonia-N adsorption by zeolite and the nitrification by biofilm in the MSL bioreactor, and further decreased ammonia-N removal from the ADSW. A similar conclusion was reported by Luo et al. (2014) [7].

**Figure 6:**
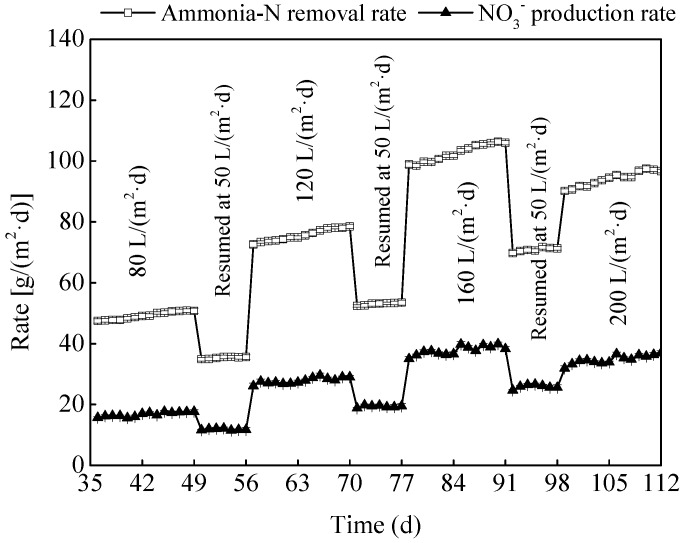
Ammonia-N removal and NO_3_^−^ production rates under different HLR (hydraulic loading rate) in the MSL (multi soil layering).

As shown in Figure 6, the trend of NO_3_^−^ production rate was similar with that of ammonia-N removal rate at different HLR in the MSL bioreactor, both increased with the increasing HLR from 80 to 160 L/(m^2^·day), indicating the occurrence of nitrification during the ammonia-N removal process. In fact, under the HLR condition of 80, 120 and 160 L/(m^2^·day), ammonia-N removal rate was appeared as 49.2, 75.7, and 102.7 g NH^+^_4_-N/(m^2^·day), respectively, and the correspondingly NO_3_^−^ production rate was showed as 16.8, 27.9, and 37.8 g NO_3_^−^-N/(m^2^·day), respectively. However, both of ammonia-N removal rate and NO_3_^−^ production rate were slightly decreased when the HLR was continue increased to 200 L/(m^2^·day), and they were shown as 94.2 g NH^+^_4_-N/(m^2^·day) and 34.8 g NO_3_^−^-N/(m^2^·day), respectively. Some other research reported quite different information that the nitrification rate was dropped obviously with the excessive increasing HLR [15,16]. On the one hand, due to the large specific surface area and ion exchange function of the zeolite in the MSL bioreactor, ammonia-N can be adsorbed more effectively than the traditional materials. On the other hand, the air layer was allowed to exist around the biofilm due to the application of media with a relatively large particle size in the MSL bioreactor, which promoted the air flow and oxygen diffusion to the biofilm, and further accelerated the mass transfer and degradation of ammonia-N [8].

#### 3.2.3. TN Removal

**Figure 7:**
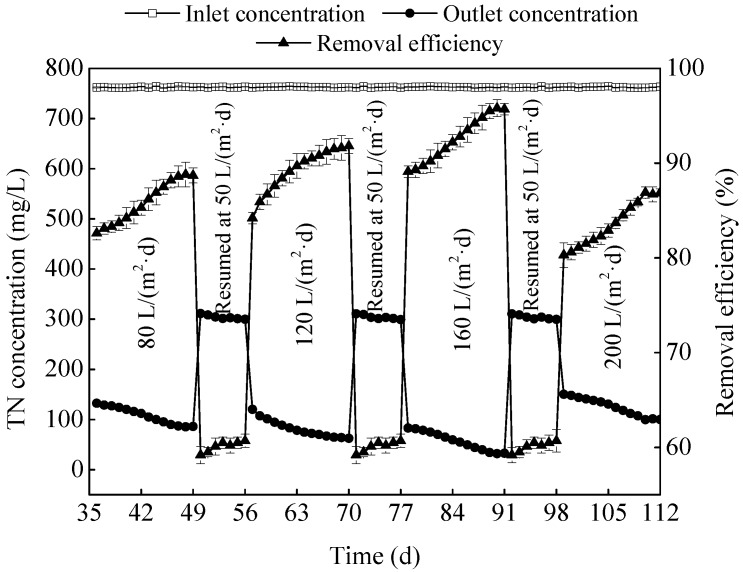
Effects of HLR (hydraulic loading rate) on TN (total nitrogen) removal by the MSL (multi soil layering).

Figure 7 showed that about 52.5–80.4% of TN was removed from the ADSW by the MSL bioreactor at different HLR, better than that by the zeolite biofilter [17], indicated that the SMB was beneficial for denitrification and thus improved TN removal from the ADSW. Denitrification was proofed by the researches on biofilm reactor as the dominant mechanism that NO_3_^−^ was converted to nitrogen gases to escape from wastewater [13].

In fact, TN removal efficiency reached 85.9%, 89.2%, and 92.5% on average at HLR of 80, 120, and 160 L/(m^2^·day), respectively, whereas 83.6% was obtained when the HLR was up to 200 L/(m^2^·day). At low HLR, the nitrogen and organic loading was low, the ammonia-N was nitrified into NO_3_^−^ and then drained out of the MSL bioreactor fast, which led to poor TN removal. This was due to the lack of carbon sources that the NO_3_^−^ could not be denitrified in time [11]. With the increasing HLR from 80 to 160 L/(m^2^·day), the denitrification rate was improved, and the TN removal efficiency was significantly increased. However, the MSL bioreactor did not perform well in TN removal from the ADSW at the excessive HLR of 200 L/(m^2^·day), due to the short HRT and the quickly shedding biofilm [18]. From the effects of HLR on TN removal, it seems that some improvements can still be made to enhance the performance of the MSL bioreactor in denitrification. Schipper et al. (2010) reported two ways to enhance the denitrification in the MSL bioreactor, one was the continuous supply of carbon source to the denitrifying bacteria, and the other was the maintenance of adequate saturated hydraulic conductivity [19]. So, alternatives for more effective denitrification of the MSL bioreactor may include: adding effective solid carbon sources into the SMBs, decreasing the particle size of the filter media in the MSL to hold more water.

## 4. Conclusions

A MSL bioreactor was constructed to remove nutrients from the ADSW, whose performance in nutrients removal was strongly affected by the HLR. Removal efficiencies of ammonia-N, TN and TP was increased with the increasing HLR from 80 to 160 L/(m^2^·day), and reached the peak values of 94.2%, 94.4%, and 92.5%, respectively, whereas the excessive HLR reduced the independent capability of the MSL bioreactor in ADSW treatment. The increasing HLR accelerated the water flow rate in the MSL, thereby accelerated the mass transfer process between the liquid phase and the biofilm phase, which can improve the degradation of pollutants, while the excessive HLR shortened the HRT and led to the quickly shedding biofilm, which limited the pollutants removal. In the ADSW treatment process, iron scraps played an important role in the TP removal, and there was only 21.4–25.8% of the TP was removed when the MSL was run with no iron addition. All in all, the MSL was effective in nutrients removal from the ADSW under different HLR.

## References

[1] Vanotti M.B., Dube P.J., Szogi A.A., García-gonzález M.C. (2017). Recovery of ammonia and phosphate minerals from swine wastewater using gas-permeable membranes. Water Res..

[2] Wen S., Liu H.Y., He H.J., Luo L., Li X., Zeng G.M., Zhou Z.L., Lou W., Yang C.P. (2016). Treatment of anaerobically digested swine wastewater by Rhodobacter blasticus and Rhodobacter capsulatus. Bioresour. Technol..

[3] Luo L., He H.J., Yang C.P., Wen S., Zeng G.M., Wu M.J., Zhou Z.L., Lou W. (2016). Nutrient removal and lipid production by Coelastrella sp. in anaerobically and aerobically treated swine wastewater. Bioresour. Technol..

[4] Zhao B., Li J., Leu S.Y. (2014). An innovative wood-chip-framework soil infiltrator for treating anaerobic digested swine wastewater and analysis of the microbial community. Bioresour. Technol..

[5] Sato K., Iwashima N., Wakatsuki T., Masunaga T. (2011). Quantitative evaluation of treatment processes and mechanisms of organic matter, phosphorus, and nitrogen removal in a multi-soil-layering system. Soil Sci. Plant Nutr..

[6] Masunaga T., Sato K., Mori J., Shirahama M., Kudo H., Wakatsuki T. (2007). Characteristics of wastewater treatment using a multi-soil-layering system in relation to wastewater contamination levels and hydraulic loading rates. Soil Sci. Plant Nutr..

[7] Luo W., Yang C.P., He H.J., Zeng G.M., Yan S., Cheng Y. (2014). Novel two-stage vertical flow biofilter system for efficient treatment of decentralized domestic wastewater. Ecol. Eng..

[8] Guan Y.D., Chen X., Zhang S., Luo A.C. (2012). Performance of multi-soil-layering system (MSL) treating leachate from rural unsanitary landfills. Sci. Total Environ..

[9] Wang L., Zheng Z., Luo X., Zhang J. (2011). Performance and mechanisms of a microbial-earthworm eco filter for removing organic matter and nitrogen from synthetic domestic wastewater. J. Hazard. Mater..

[10] Sato K., Masunaga T., Wakatsuki T. (2005). Water movement characteristics in a multi-soil-layering system. Soil Sci. Plant Nutr..

[11] Guo J.Y., Zhou Y.L., Jiang S.L., Chen C. (2018). Feasibility investigation of a multi soil layering bioreactor for domestic wastewater treatment. Environ. Technol..

[12] American Public Health Association, American Water Works Association, Water Environment Federation (1998). Standard Methods for the Examination of Water and Wastewater.

[13] Zhang Y., Cheng Y., Yang C.P., Luo W., Zeng G.M., Lu L. (2015). Performance of system consisting of vertical flow trickling filter and horizontal flow multi-soil-layering reactor for treatment of rural wastewater. Bioresour. Technol..

[14] Krasnits E., Beliavsky M., Tarre S., Green M. (2013). PHA based denitrification: Municipal wastewater vs. acetate. Bioresour. Technol..

[15] Hu B., Wheatley A., Ishtchenko V., Huddersman K. (2011). The effect of shock loads on SAF bioreactors for sewage treatment works. Chem. Eng. J..

[16] Van den Akker B., Holmes M., Pearce P., Cromar N.J., Fallowfield H.J. (2011). Structure of nitrifying biofilms in a high-rate trickling filter designed for potable water pre-treatment. Water Res..

[17] Guo J.Y., Zhou M.J., Gan P.F., Tan X.D., Guo Z.H., Fu L., Huang W.Y., Bai X. (2016). Performance of zeolite trickling filter in treatment of domestic wastewater and characteristics of the biofilm. China Environ. Sci..

[18] Healy M.G., Rodgers M., Mulqueen J. (2006). Denitrification of a nitrate-rich synthetic wastewater using various wood-based media materials. J. Environ. Sci. Health Part A.

[19] Schipper L.A., Robertson W.D., Gold A.J., Jaynes D.B., Cameron S.C. (2010). Denitrifying bioreactors—An approach for reducing nitrate loads to receiving waters. Ecol. Eng..

